# The role of complement in the tumor microenvironment

**DOI:** 10.12703/r/10-80

**Published:** 2021-11-29

**Authors:** Danyaal Ain, Talha Shaikh, Samantha Manimala, Berhane Ghebrehiwet

**Affiliations:** 1 The Department of Medicine, Stony Brook University, 100 Nicholls Road, Stony Brook, NY 11794-8161, USA

**Keywords:** complement, activation fragments, gC1qR

## Abstract

Tumorigenesis has long been linked to the evasion of the immune system and the uncontrolled proliferation of transformed cells. The complement system, a major arm of innate immunity, is a key factor in the progression of cancer because many of its components have critical regulatory roles in the tumor microenvironment. For example, complement anaphylatoxins directly and indirectly inhibit antitumor T-cell responses in primary and metastatic sites, enhance proliferation of tumor cells, and promote metastasis and tumor angiogenesis. Many recent studies have provided evidence that cancer is able to hijack the immunoregulatory components of the complement system which fundamentally are tasked with protecting the body against abnormal cells and pathogens. Indeed, recent evidence shows that many types of cancer use C1q receptors (C1qRs) to promote tumor growth and progression. More importantly, most cancer cells express both C1q and its major receptors (gC1qR and cC1qR) on their surface which are essential for cell proliferation and survival. In this review, we discuss the ability of cancer to control and manipulate the complement system in the tumor microenvironment and identify possible therapeutic targets, including C1q and gC1qR.

## Introduction

### What is cancer? 

Cancer can be defined as the autonomous growth of transformed cells, which eventually leads to the formation of masses called tumors. Generally, healthy cells have checkpoints and mechanisms to maintain their cycle of growth and performance. Cancer cells, however, have lost the ability to stop growing when necessary and this can be quite dangerous given that cells endure non-beneficial genetic changes over their lifetime^1^. Three distinct features of cancer are immune invasion, angiogenesis, and metastasis. These hallmarks can be expanded and compartmentalized into features such as proliferative signaling, growth suppressors evasion, apoptosis or programmed cell death resistance, angiogenesis, and metastasis or invasion activation^2^. In order to enhance their growth and survival, tumor cells can promote the progression of the cancer by using a tumor-associated stroma, which in turn consists of recruited cells and host cells of the infiltrated tissue. Multiple stromal cell types, along with other cells and proteins in the environment of the tumor, make up the tumor cell microenvironment, which essentially sustains and protects the growing cancerous mass^2^. Complement proteins interact with proteins in the tumor cell microenvironment and play a role in many of the hallmarks of cancer, such as immunosuppression, angiogenesis, inhibition of apoptosis, and effective immune evasion.

## The role of complement in immunity

**Figure 1.**
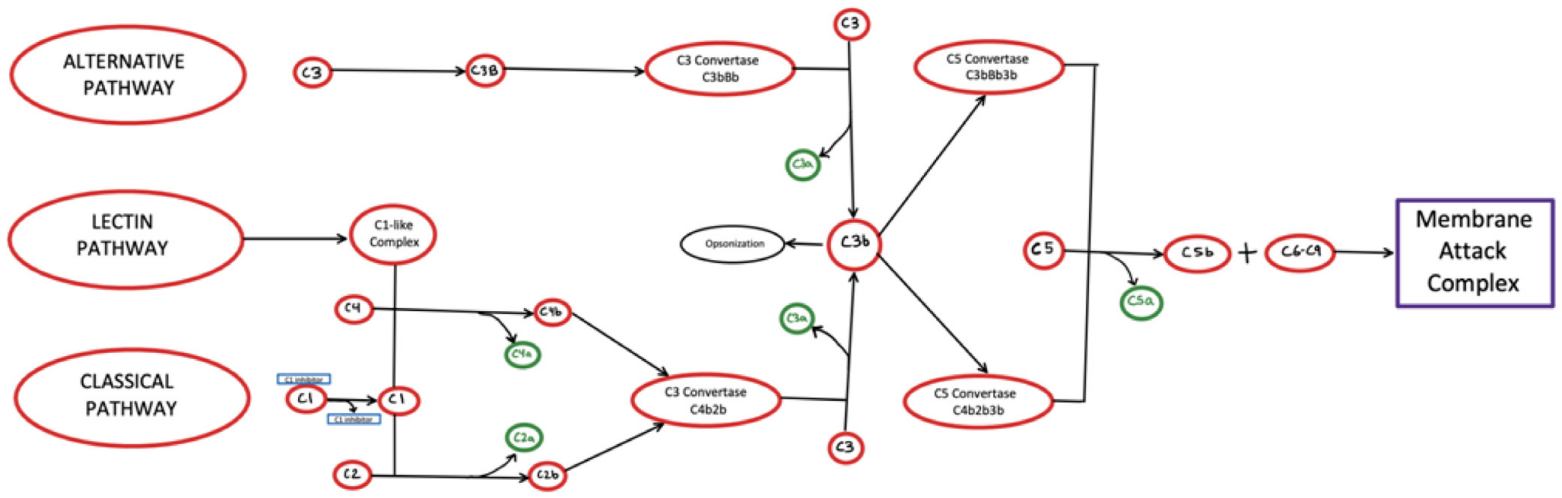
A model of the complement system, including the different pathways. The classical pathway is activated via recognition of C1q to antigen-bound antibody constant regions. The alternative pathway is activated via a “tickover” process in which low-level hydrolysis of complement C3 initiates C3 convergence. Lastly, the lectin pathway is activated by either mannose-binding lectin or ficolins. As seen above, all three pathways converge on C3, ultimately allowing for the development of the common terminal pathway, producing membrane attack complex, which is then responsible for producing an osmotic imbalance in target cells, eventually leading to cell lysis.

The complement system is a well-orchestrated and highly regulated biological system and consists of three independent but interactive pathways (Figure 1). Although each pathway is activated by target-specific antibodies or by structural elements on the target cell or pathogen, they all converge at the level of C3, which is cleaved by C3 convertase to generate C3b. Once the C3b is membrane-bound, the sequential recruitment and activation of C5 followed by the assembly of the terminal complement proteins C5b–C9 complex or membrane attack complex (MAC) are common to all three pathways. Once MAC is assembled on the pathogen or cell surface, the target cell is destroyed by osmotic lysis in a manner similar to the cell lysis caused by the pore forming cytolytic protein perforin, which is found in the granules of cytotoxic T lymphocytes and natural killer cells.

The classical complement pathway is activated when antibody–antigen complexes are formed on bacterial or cell surfaces. C1q, which circulates in plasma as part of a calcium-dependent pentamolecular complex, together with two molecules of C1r and two molecules of C1s, then binds to the constant region of the antigen-bound antibody (IgM or IgG)^3^. The binding of IgM to an antigen then causes a conformational change, within the pentameric C1 protein thereby exposing C1q-binding sites that otherwise are hidden^3^. Although, IgG, like IgM has also C1q-binding sites, it requires specific binding density to achieve IgM-like affinity with C1q^3^. The binding of C1q in turn induces a conformational change in the pentamolecular complex, leading to activation of C1r followed by activation of C1s^4^. C1s subsequently cleaves C4 into C4a, which diffuses away, and C4b, which binds via a thioester bond to the cell surface^3^. Complement C2 recognizes the cleaved C4b complement and binds to it, allowing further cleavage by C1s to produce C2a and C2b. The C2b then remains bound to C4b and causes the assembly of an enzymatically active multi-protein complex: the C3 convertase (C4bC2b). Cleavage of C3 by C3 convertase in turn generates two fragments: C3a and C3b. C3a is an anaphylatoxin, which can trigger inflammation, cell migration, and activation. The C3b fragment on the other hand binds to C4bC2b to form the C5 convertase^3^. In a manner similar to C3, C5 then gets cleaved to produce the anaphylatoxin C5a, and C5b. Like C3a, C5a promotes inflammation by increasing vasodilation and vascular permeability and releases pro-inflammatory mediators through the activation of granulocytes and macrophages^5^. C5b subsequently interacts sequentially with C6, C7, C8 and C9 to form the C5b-9 complex or MAC, an effector protein that directly forms transmembrane channels on the surface of pathogen cell membranes and is responsible for pore formation and eventual cell lysis^3^.

The alternative complement pathway is activated by bacterial and viral structures, specifically bacterial endotoxin, which is a lipopolysaccharide on the outer membrane of Gram-negative bacteria. The activation of the alternative complement pathway occurs via a process termed “tickover” of C3, a spontaneous low-level hydrolysis of C3 to form C3(H_2_O). C3(H_2_O) then acquires the ability to bind to factor B, which induces a conformational change in factor B, making it susceptible for cleavage by factor D, thus generating Ba and Bb fragments^6^. The Bb fragment remains bound to the initial complex and self-regulates the cleavage of numerous C3 molecules to produce C3b. C3b proceeds to interact with factor B, resulting in the generation of the initial alternative pathway C3 convertase. Another mechanism of activation of the alternative complement pathway is through properdin. Properdin is a gamma globulin protein, which binds to C3b and subsequently activates factor B and factor D, resulting in the formation of C3bBb, a C3 convertase^5^. The rest of the function of the alternative complement pathway is similar to that of the classical pathway in that C3 convertase promotes the development of the C5 convertase, which then produces the MAC.

The lectin pathway of the complement system of humans is activated by mannose-binding lectin (MBL) or one of the three types of ficolins (L-ficolin, M-ficolin, and H-ficolin), recognizing and binding to patterns of sugars or acetyl groups on the surfaces of microorganisms^7–10^. MBL-associated serine protease (MASP) dimers form complexes with either MBL or ficolin that upon binding to pathogens cause a conformational change in MASP^11,12^. The MASP, often MASP-1 or MASP-2, cleaves and activates another MASP in the same complex^12^. The activated MASP then cleaves complement proteins C2 and C4, which respectively release the fragments C2a and C4b^13^. These two fragments associate with each other and form the lectin pathway C3 convertase, C4b2a^13^. Much like in the classical and alternative pathways, C4b2a cleaves C3 into C3a, which is released to initiate a local inflammatory response, and C3b fragments, which covalently bind to pathogen surfaces and interact with C4b2a to make the C5 convertase^14^. Additionally, pathogens bound to C3b can activate the alternative pathway and are engulfed by phagocytes expressing receptors for C3b^13^. C5b, formed from the cleavage of C5, then initiates the formation of MAC in a manner similar to the classical pathway^13^.

Because of its potential to lyse and destroy normal bystander cells, each step of the activation is tightly regulated by specific enzymes. Therefore, the complement system not only constitutes a powerful arm of innate immunity but also serves as a bridge between innate and adaptive immunity. Discovered more than a century ago as a system that “complements” the function of antibodies in the process of phagocytic elimination of pathogenic microorganisms from the site of infection, it is now regarded as a system involved in a myriad of immunological functions that can be both protective and destructive to the organism. It is not surprising, therefore, that tumor cells have developed many methods of not only protecting themselves from its destructive power but employing complement proteins to also enhance their own survival and growth. The significance of complement in health and disease is further demonstrated by the fact that deficiency in complement components such as C1q, C2, and C4, though uncommon, is associated with a diverse array of pathological disorders, including autoimmune diseases and susceptibility to infections. Deficiency in C3, for example, is very rare as it can lead to severe infections and other inflammatory diseases. Similarly, knocking out MBL in mice was shown to lead to an increased susceptibility to infection, abnormal inflammatory responses and coagulation, and protection from ischemia-reperfusion injuries^10^. Additionally, asparaginase, used in the treatment of acute lymphoblastic leukemia, has been shown to inhibit the lectin pathway of the complement system by reducing its effect on levels of mannan-bound MBL/MASP-1 and MBL/MASP-2 complexes^15^. Moreover, knocking out ficolin, which has a role similar to that of MBL in the lectin pathway, has also been shown to increase susceptibility to infection in mice^10^.

## The role of the complement system in tumor progression

Tumor cells have been shown not only to synthesize selected complement proteins but also to express and display receptors for complement proteins^16^. For example, plasma concentrations of C3 and C5a were shown to increase in mice with metastatic breast cancer, and blockade of the C5a receptor (C5aR) reduced lung metastases^16^. C5aR has also been shown to inhibit recruitment and function of CD4^+^ and CD8^+^ cells and to suppresses T-cell responses in organs where tumors have metastasized in these mice^16^. Although silencing the C3 and C5 in mice with ovarian cancer reduces tumor growth, it has been proposed that this reduction is independent of T cells^17^ and is likely due to the action of another complement protein. Levels of C4d, a fragment of complement protein C4b generated in the classical pathway, are elevated in patients with lung cancer^18^ and patients with oral and oropharyngeal squamous cell carcinoma^19^.

Complement proteins also interact with tumor-associated macrophages (TAMs) and tumor-associated neutrophils, both of which contribute to tumor progression. TAMs have been shown to non-specifically promote growth and metastasis of malignant metastatic tumors *in vitro*, directly interact with cancer cells, promote angiogenesis, and suppress adaptive immunity^20,21^. Although TAMs are present in the tumor microenvironment (TME) and are associated with tumor progression, their role is unclear^22^. C1q can induce polarization of macrophages, which express increased levels of programmed death-ligand 1 (PD-L1) and PD-L2 as well as suppressed surface CD40 and suppressed proliferation of inflammatory T cells after polarization^23^. Other complement fragments capable of inducing macrophage polarization include C5a and C5aR, the latter of which is expressed on TAMs and plays a role in the regulation of the M2 phenotype of TAMs^24^. C5a also stimulates the release of leukotriene B4 from epithelial and endothelial cells, which helps facilitate neutrophil recruitment^25^. Furthermore, inappropriate activation of the complement system and deposition of complement proteins have been observed as common characteristics of various tumors^26^. This activation in turn is postulated to contribute to tumor cell proliferation, migration, invasion, and epithelial–mesenchymal transition. In particular, the complement system is responsible for the introduction of potent anaphylatoxins that have been linked to various effects on the TME-promoting pathways directed toward immune evasion^27^.

## Complement and the adaptive immune response

Immune invasion is a hallmark of cancer and allows cancer cells to survive and proliferate explosively under disguise. The complement system plays a large part in the ability of cancer to circumvent the immune system. Therefore, it is important to examine the complement activity in the TME. The production of antibodies, lysis of pathogens, B-lymphocyte activation, and opsonization are some of the many critical functions of the immune system which are facilitated by and dependent on the complement system. Many complement proteins such as MBL, ficolins, and most importantly C1q play pivotal roles in pathogen recognition^28^. The intertwined nature of the complement and immune system makes the former the perfect target for cancerous cells to take advantage of in their quest for immune evasion.

A recent study on C1q and its receptors showed that the proliferation of the breast cancer cell line SkBr3 is inhibited by addition of either exogenous C1q or its globular head domains, presumably by blockade of surface gC1qR-dependent pro-proliferative signaling^29^. Since actively dividing tumor cells are known to overexpress as well as release gC1qR into the tumor cell microenvironment, it is postulated that the secreted gC1qR serves either as an autocrine signal for cancer cell proliferation or as a molecular shield protecting the tumor cell from C1q-induced tumor cell destruction^29^. Indeed, exogenous C1q has been shown to activate p38 and caspase 3 to induce apoptosis via autophagy. Furthermore, ADAM28 (a disintegrin and metalloproteinase 28) is overexpressed in certain carcinomas and actually reduces C1q-induced cytotoxicity^30^.

Clq is a versatile regulatory complement protein for cancer in that it can promote proliferation when bound to gC1qR and cause cell apoptosis depending on where the interaction is taking place in the TME. Human T cells of the adaptive immune system are known to express C1q and its receptors and can induce anti-proliferative, apoptotic signals involving the proliferation/activation of T cells. Specifically, C1q in the TME can suppress CD4^+^ T cells by binding to the gC1qR in a manner similar to that of the PDL-1 and PD-1 axis^31,32^. As a modified self, a cancer cell perforce expresses proteins that induce antibody production. These “autoantibodies” in turn have the potential to bind to the tumor cell antigen forming a suitable target for C1q binding and complement activation resulting in MAC-mediated destruction. This phenomenon, however, never happens as the tumor cell microenvironment is rich in molecules that collectively serve as a molecular shield preventing this from happening. Included among these molecules are various complement proteins (such as C3, C3a/C3aR, C5a/C5aR, and C1q) and membrane-bound complement regulatory proteins (mCRPs), both of which have increased expression in many malignant tumors and cancer cell lines^33^. There is also evidence that overexpression of mCRPs in cancer cells may protect them from complement-mediated attack by MAC and eventual cell lysis^26,34^. One of these molecules is secreted gC1qR, which (as mentioned earlier) can bind to C1q and activate the classical pathway in the pericellular rather than on the tumor cell surface. In addition, soluble gC1qR can bind and activate the kinin system, thereby generating activation fragments such as bradykinin—a potent vasoactive peptide which can induce vascular permeability and thus enhance cancer cell metastasis. Thus, by simultaneous activation of the complement system and the kinin system, activation fragments that collectively contribute to tumor cell survival, proliferation, and metastasis are generated^29^.

## Complement and immunosuppression

Among the many hallmarks of cancer, immune evasion is one that contributes to its deadly and elusive properties. Mechanisms of cancer allow for the perversion of proto-oncogenes and the silencing of tumor suppressors that otherwise would allow the body to effectively contain and prevent a tumor growth. Immune evasion and the blindsiding nature of cancer can be effects of “immune editing”, in which tumors suppress T-cell function, anti-cancerous secretions and immune system mediators^35^. One of the pivotal components of the TME is the myeloid-derived suppressor cell (MDSC), which have been shown to assist in tumor evasion from responses of the immune system, promote tumor resistance to immunotherapy, and sustain tumor survival and progression due to suppression of T cell-dependent tumor cytotoxicity through mechanisms involving production of nitric oxide and cytokines^36^. Studies have provided evidence for a correlation between the recruitment and activation of MDSCs in tumors with complement activation via C5a^3^. Specifically, C5a has been identified as a chemoattractant for a subsect of MDSCs that produce and release reactive oxygen and nitrogen species for efficient immunosuppression^3^. Additionally, C5a has been found to have a profound influence on the functional capability of MDSCs in suppressing CD8^+^ T cell-mediated anti-tumor responses, as shown by experiments in which isolated MDSCs from C5aR-deficient mice were unable to suppress the proliferation of T cells^25^. Thus, the recruitment of MDSCs into the tumor site by C5a is critical in generating an immunosuppressive microenvironment through the inhibition of T cell-mediated anti-tumor responses.

## Complement and angiogenesis

Angiogenesis, the formation of new blood vessels within the tumor mass, is a critical aspect of tumor survival and growth. For tumors to continue growing in size, they require nutrition, oxygen, and waste removal like normal tissues. The only way that cancer cells, deeply lodged in tumor tissue, can receive these critical necessities is through the formation of new blood vessels or angiogenesis. Although angiogenesis is known to occur during wound healing and the reproductive cycle, it is not active at all times. Therefore, tumors hijack and use this regulated, ordinary bodily function to continuously grow and expand^37^. The new vascular networks generated during angiogenesis additionally assist the proliferation and metastatic spread of cancer cells. Studies conducted to examine the relationship between complement components and angiogenesis have found both pro-angiogenic and anti-angiogenic roles in neovascularization^3^. Examination of epithelial ovarian cancer in genetically C3-deficient and C5aR knockout mice demonstrated dysregulated endothelial cells, impairing tumor vascularization as well as altered vascular endothelial growth factor (VEGF) expression^38^. VEGF is a key cytokine for tumor metastasis and its concentration has been found to be directly proportional to malignancy^39^. However, C3-deficient and C5aR-deficient mice were found to have increased pathological retina neovascularization, indicating that the absence of these complement proteins resulted in increased postnatal angiogenesis^40^. This dual function of complement in turn suggests that complement activation may be relevant only in the context of initial tumor formation. Additionally, mice with homozygous C1q deficiency were shown to have defective vessel formation that was restored to normal by locally applying C1q^41^.

## Complement and inflammation

Several studies have suggested that tumor-promoting inflammation is a considerably pivotal component of the TME and is responsible for the reciprocal promotion of tumor growth. Among the hallmarks of cancer-related inflammation are TAMs, the presence of pro-inflammatory cytokines and chemokines, and the infiltration of white blood cells^42^. As mentioned earlier, C5a is a potent anaphylatoxin, which induces inflammation through its ability to stimulate the release of histamine from mast cells^39^. It has been shown that C5a signaling results in the increased expression of interleukin 6 (IL-6), which is responsible for responding to infections and tissue injuries and whose dysregulated synthesis can have pathological effects on chronic inflammation and autoimmunity^43^. The veracity of this concept was shown in a mouse model of lung carcinogenesis in which blocking C5aR signaling resulted in the downregulation of IL-6 expression^5^. Clinical studies have demonstrated that increased levels of serum IL-6 have been associated with advanced tumorigenesis in various cancers, including multiple myeloma, non-small cell lung carcinoma, colorectal cancer, renal cell carcinoma, breast cancer, and ovarian cancer^5^. Additionally, local complement activation in the TME contributes to the inhibition of tumor-infiltrating lymphocytes (TILs), which are responsible for IL-10 production^44^. In a murine model of melanoma, it was shown that the ability of CD8^+ ^TILs to secrete IL-10 was inhibited by C3a and C5a^44^. Inhibition of IL-10 in the presence of tumorigenic activity in turn results in unregulated inflammation, thereby impeding pathogen clearance and mitigating immunosuppressive functions^45^.

## Complement and inhibition of apoptosis

To combat rampant cell proliferation, tumor suppressor genes in the genome, induce apoptosis in certain cases such as aging cells, abnormally divided cells, and genetically damaged cells. In cancer, however, tumor suppressors are inactivated, which allows cancerous cells to continue proliferating^46^. Two well-studied tumor suppressor genes are *RB* (retinoblastoma), which is mutated in retinoblastoma, and *TP53*, which is most commonly mutated in human cancers^47^. Many downstream products of oncogenes can have pro-tumor activities, and the stress from this oncogenic signaling along with DNA damage is a cue for apoptosis to occur. If tumor suppressors such as TP53 are silenced, apoptosis cannot happen. This inactivation, combined with proliferative signaling from oncogenes, is a leading cause of tumorigenesis. Thus, the cell damage-sensing capabilities of TP53 are critical for the prevention of cancer^2^.

**Figure 2.**
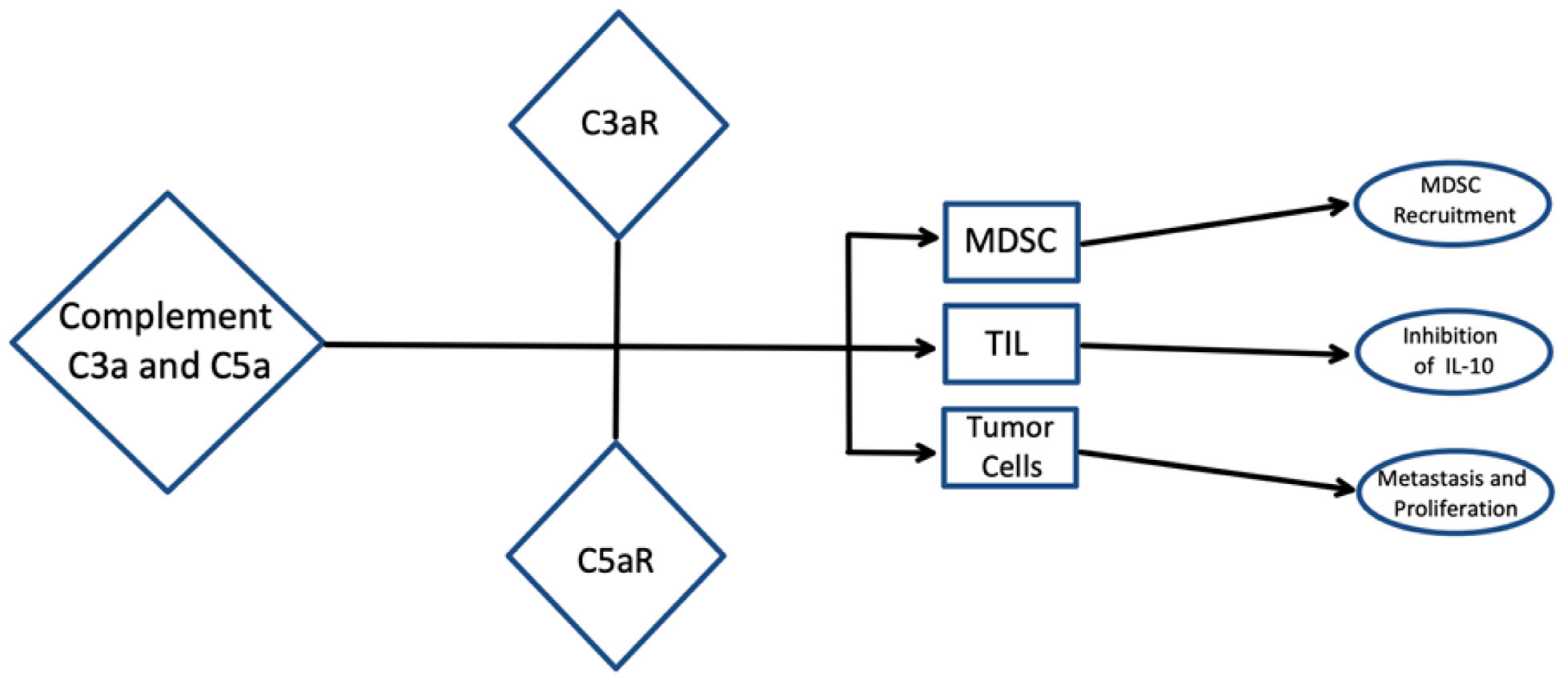
Complement and inhibition of apoptosis. A model displaying the role of complement activation, specifically via complement proteins C3 and C5, in the tumor microenvironment. The complement system promotes the release of C3a and C5a, which are responsible for tumor progression. C5a is a chemoattractant for certain myeloid cells, including myeloid-derived suppressor cells (MDSCs), into the tumor. MDSCs are responsible for producing and releasing certain oxygen and nitrogen species, resulting in immunosuppression. C5a has also been shown to suppress CD8^+^ tumor-infiltrating lymphocytes (TILs) which in turn reduces interleukin-10 (IL-10) production, ultimately promoting inflammation and inhibition of pathogen clearance. Complement proteins can also develop an autocrine loop in which cell proliferation and additional metastasis promotion are presented. Thus, C3a and C5a have been shown to mediate complement activation on MDSCs, TILs, and cancer cells.

Recent studies have suggested that complement activation may promote tumor progression via anti-apoptotic mediators (Figure 2). C5a has been shown to prevent apoptotic caspase 3 activation and DNA fragmentation^39^. Additionally, the binding of C5a to C5aR has led to activation of the mitogen-activated protein kinase (MAPK) pathway, which has inhibitory effects on glutamate-induced apoptosis^39^. It was also observed that C3 inhibition resulted in increased basal and cytokine-induced apoptosis in beta cells and that the addition of exogenous C3 prevents this apoptosis^48^. C1q binds to surface blebs on apoptotic keratinocytes and apoptotic vascular endothelial cells via its globular head domains and can initiate uptake of apoptotic cells into macrophages^49–51^. Defects in complement-dependent clearance of apoptotic cells have been associated with increased susceptibility to autoimmunity development^52^. For example, C1q- and C4-deficient mice were shown to have impaired clearance of apoptotic cells, resulting in the development of a lupus-like disease^48^. One study showed that mice with homozygous C1q deficiency develop glomerulonephritis associated with multiple apoptotic bodies and that the mice that did not develop glomerulonephritis had significantly higher levels of glomerular apoptotic bodies^53^. As previously stated, the role of complement in tumor progression is twofold and is dependent on the specific TME. Therefore, understanding of the complement components in the tumor cell microenvironment and the conditions that promote or enhance tumorigenesis and tumor survival is critical.

## C1q and its role as a cancer-promoting factor

**Figure 3.**
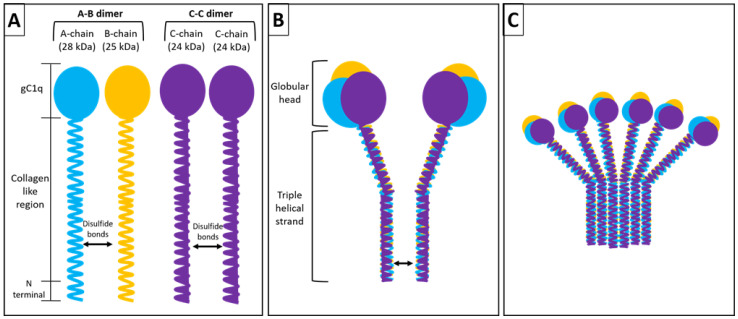
Structure of C1q. The intact C1q molecule (460 kDa) is assembled from 18 similar but distinct individual chains (6A, 6B, and 6C). The chains are organized to form 6 individual strands each of which consists of a single A (28 kDa), B (25 kDa) and C (24 kDa) chains. The three chains are the product of three distinct genes which are highly clustered and aligned 5’ to 3” in the same orientation in the order ACB on a 24 kb stretch on chromosome 1p. In each strand, the A chain is disulfide linked to the B chain, whereas the C chain is disulfide linked to the C chain of a neighboring strand to form a doublet. Three doublets are associated by strong non-covalent forces to give the intact C1q molecule its characteristic hexameric and “bouquet of tulips” like electron microscopic structure^62^.

The first component of complement C1q is unique in that it is predominantly synthesized extrahepatically by macrophages, dendritic cells (DCs), endothelial cells, fibroblasts, and microglial cells. It is a 460-kDa hexameric glycoprotein with six collagen-like regions and six globular heads (Figure 3)^54,55^. Each unit of this hexamer consists of polypeptide chains A (28 kDa), B (25 kDa), and C (24 kDa) that form a collagen-like triple-helical strand between their N terminal regions and form a globular head from their C terminal regions^56,57^. Additionally, there are disulfide bonds between chains A and B within a strand as well as between the C chains of two adjacent strands, thus forming a doublet^58^. C1q is the recognition subunit of the classical pathway complex C1^59^. Recent experimental evidence shows that in addition to playing a role in triggering the classical pathway of complement activation and phagocytosis, C1q plays a significant role in cancer^60,61^.

The fact that C1q induces angiogenesis indicates that it plays a major role in tumor growth^41^. In prostate cancer, for example, C1q has been shown to destabilize cell adhesion by activating the tumor suppressor WOX1, thereby blocking cancer cell proliferation and induction of apoptosis^63^. In a mouse model of melanoma, it was shown that those that were C1q-deficient were found to have reduced tumor mass, prolonged survival, and fewer lung metastases in comparison with wild-type mice^64^. C1q is also expressed in the TME on vascular endothelial cells, spindle-shaped fibroblasts, and monocytoid cells^64^.

Complement C1q has been shown to mediate various immunoregulatory functions, including enhancement of phagocytosis, regulation of cytokine production in antigen-presenting cells, and enhancement of T-lymphocyte maturation^65^. Recent studies also have shown that C1q can enhance cancer development and proliferation. Indeed, the level of C1q is increased within the TME of most cancer cells and is also^64^ synthesized within the TME by macrophages and DCs to enhance pro-tumorigenic activity, including angiogenesis^66^. Therefore, the local synthesis of C1q provides strong evidence for the observed C1q-mediated functions that are independent of complement activation.

C1q has been identified in the stroma and vascular endothelium of several human malignant tumors, including colon, lung, breast, pancreatic carcinoma, and melanoma^65^. This abnormal presence indicates that the activation of the classical pathway via C1q can be related to complement-mediated tumor progression. Bulla *et al*. showed an increased deposition of C1q and associated augmentation of vascular density and lung metastases in wild-type mice in comparison with C1q-deficient (C1qa^−^/^−^) mice^64^. Additionally, it was observed that the C1q-deficient mice showed stagnant tumor growth and extended survival^64^. These findings support the premise that locally synthesized C1q is a tumor-promoting factor and enhances many processes that favor tumor progression, including cell adhesion, migration, proliferation angiogenesis, and metastasis^66^.

Agostinis *et al*. examined the role of C1q in the microenvironment of malignant pleural mesothelioma (MPM) and identified that C1q bound to hyaluronic acid (HA) via its globular domain subsequently acquired pro-tumorigenic properties, inducing adhesion, migration, and proliferation of mesothelioma cells^67^. HA has also been identified to promote proliferation and migration of MPM cells via binding to the hyaluronan receptor. However, HA-bound C1q showed increased adhesion of mesothelioma cells in comparison with independent HA. This finding suggests that C1q does indeed have a pro-tumorigenic effect on mesothelioma cells, a property that appears to be a factor common to various other tumors. Additionally, HA-bound C1q was shown to promote MPM tumor progression by enhancing mesothelioma cell proliferation and migration in comparison with HA or C1q alone.

Other studies have suggested that C1q has a binary function in relation to cancer, having both pro-tumorigenic and anti-tumorigenic properties. For example, in the MPM environment, C1q was shown to implement a defensive response against apoptosis, thereby enhancing tumorigenesis^67^. However, C1q was also shown to induce apoptosis and mitigate the growth of human prostate DU145 cells^63^. Ultimately, the degree of complement activation has been determined to be dependent on the specific tumor type as well as the degree of inflammation corresponding to tumor proliferation. Certain tumors have been shown to reduce the cellular response to C1q by decreasing the number of C1q receptors on the cell surface in an attempt to restrain apoptosis induction^66^. Since there are two types of C1q receptors, one recognizing its globular heads (gC1qR) and the other binding to its collagen tails (cC1qR), the orientation of the bound C1q molecule (that is, heads versus tails) may explain the various C1q-mediated functions.

## C1qRs and their roles in cancer

The two receptors for C1q—cC1qR/CR and gC1qR—are highly upregulated on many types of tumors and may have different functions in tumor cells^20^. Blockade of these receptors with monoclonal antibodies (mAbs) has been shown to inhibit tumor growth, suggesting that these receptors play a role in tumor progression^68^.

cC1qR/CR is a 60-kDa membrane protein that binds predominantly to the collagen region of C1q and is identical to calreticulin (CR)^69,70^. Its structure reveals three beta sheets and three hydrophobic clusters^71^. In addition to binding to C1q, cC1qR binds to mannan-binding protein, conglutinin, and lung surfactant protein, all of which have collagen domains^72^. cC1qR/CR has been shown to mark malignant cells for destruction by macrophages^20^. Phagocytosis of apoptotic cells by human monocyte-derived macrophages has been shown to be effectively inhibited by anti-cC1qR antibodies^51^. Indeed, cC1qR/CR-induced phagocytosis of tumor cells was shown to be suppressed by blocking or knocking down cC1qR/CR as well as by the increased expression of CD47 on malignant cells^73,74^. cC1qR has also been shown to bind to the collagen tails of C1q and MBL attached to apoptotic cells^51^.

Another receptor for C1q is gC1qR—also referred to as p33, p32, and HA-binding protein (HABP1)—which binds to the globular heads of C1q^75^. Its crystal structure reveals that it is a homotrimer with two differentially charged faces: a highly charged face called the solution face (or S-face) and a less charged one called the membrane face (or M-face). Each subunit of the trimer is composed of a seven-strand antiparallel beta sheet and three alpha helices^75^. The S-face of the trimer contains the C1q-binding site^76^. gC1qR is a ubiquitously distributed multi-functional and multi-compartmental molecule, and is unique in that it contains both an ITAM (immunoreceptor tyrosine-based activation motif) and an ITIM (immunoreceptor tyrosine-based inhibition motif)^77^. It is abundantly and ubiquitously expressed on the surface of a wide range of cell types including endothelial cells, platelets, and T cells as well as in the mitochondrial matrix. Experimental evidence accumulated to date shows that gC1qR can interact with various molecules in the tumor cell environment, including proteins of the kinin system such as factor XII and high-molecular-weight kininogen (HK), thereby triggering activation of the kinin–kallikrein system (KKS) resulting in the generation of bradykinin—one of the most potent vasoactive peptides known^78^.

The binding of gC1qR to inflammatory molecules indicates its role in tumor progression due to the large influence of inflammatory cells on the TME^79^. It has been shown that the addition of mAb that blocks the C1q-binding site of gC1qR inhibits cell proliferation^80^. Moreover, proliferating tumor cells secrete gC1qR into the pericellular milieu as a defense mechanism to prevent the tumor cell from C1q-mediated cellular damage^20^. Because surface-expressed as well as secreted gC1qR is crucial for tumor cell survival, it has become a potential target for the development of anti-tumor therapy^78^. Although (by virtue of its ubiquitous expression) an off-target effect using antibody or small molecule-based therapy is indeed of concern, it is possible to target only the antigenic site responsible for the anti-tumor effect without inhibiting other sites that are critical for other biologic functions.

## The C1q–gC1qR axis as a potential novel checkpoint inhibitor

The gC1q-binding site on gC1qR is covered by residues 76 to 93 located on the gC1qR S-face^73,81^ and this interaction has been shown to activate the classical pathway of complement. More pertinently, the addition of C1q to mitogen-induced proliferating T cells has been shown to inhibit cell proliferation^82,83^. Similarly, the addition of exogenous C1q to breast cancer cells in culture was shown to have an anti-proliferative effect whereas the addition of recombinant gC1qR was pro-proliferative^16^. Since C1q, cC1qR, and gC1qR are expressed by breast cancer cells and have roles in cancer cell proliferation, the C1q–gC1qR axis and the C1q–cC1qR axis represent potential new targets for the development of anti-cancer therapy^16^.

The C1q–gC1qR interaction in the TME appears to mimic that of the PD-1/PD-L1 interaction with the cancer cell surface-expressed C1q serving the role of PD-L1 and the immune cell-expressed gC1qR serving the role of PD-1, thereby attenuating the T-cell signals directed at attacking the tumor cells^19^. PD-1 is expressed on many immune cells, such as natural killer cells, monocytes, activated T cells, and B cells and even weakly on immature T cells and B cells^84,85^. Its ligand, PD-L1, is expressed by a variety of cells, including lymphocytes, monocytes, and lung cells, and is upregulated in tumor cells^86,87^. The binding of PD-1 to PD-L1 activates intracellular signaling pathways and inhibits immune cell activation^84^. The PD-1/PD/L1 axis can also suppress T-cell proliferation by inhibiting the Ras/MEK/ERK pathway^88^. The C1q that is expressed on tumor cells is anchored via a membrane-binding domain located on the A-chain, thereby exposing the six globular heads outwardly to bind to as many as six antigens simultaneously. This configuration therefore would be extremely efficient in binding to multiple gC1qR molecules expressed on the T cells or other immune cells in the TME, thereby inhibiting their anti-tumor functions and enhancing uninhibited tumor cell growth.

## gC1qR as a marker of cell proliferation

The complement system contains many components that are involved in the TME and one of its key factors is the multi-functional gC1qR. In addition to being found in various compartments of the cell, gC1qR has been shown to be an important modulator of extracellular and intracellular ligands. In oncogenesis and the development of cancer, gC1qR is of great interest as it appears to be upregulated in various adenocarcinomas^75^.

Evidence accumulated to date suggests that gC1qR potentially serves as a marker of cell proliferation as it is overexpressed in most epithelial tumors. The study, by Dembitzer *et al*., highlighted the changes in gC1qR expression among various types of tumor tissues^78^. Employing UltraVision LP Detection System (Thermo Fisher Scientific Inc., Waltham, MA, USA) and the mAb 60.11 to gC1qR, Dembitzer *et al*. showed expression of gC1qR in both mesenchymal and epithelial tissue that was malignant, normal, inflammatory, or dysplastic. Staining techniques and light microscopy yielded results showing that mesenchymal tumors had weaker gC1qR expression than epithelial tumors. Epithelial tumors, normal cells that were proliferating, and benign inflammatory lesions were noted to have the strongest gC1qR expression^78^. The results of this study are highly suggestive that gC1qR plays a role in the proliferation of certain tumorigenic cells and its elevated levels may indicate malignant tissue growth.

It is known that membrane-bound gC1qR can bind to the globular heads of C1q and thereby trigger classical pathway complement activation. The interaction of C1q with membrane-bound gC1qR is therefore vital for innate immune response to infection of cells^89–91^. Recent experiments have provided evidence that soluble gC1qR that is secreted into the TME serves as a proliferative autocrine signal^16^. The study used the SkBr3 Her2^+^ breast cancer cell line that expresses cC1qR, gC1qR, and C1q. When the cells were cultured with anti-Clq that binds to membrane-bound C1q, cell growth was completely inhibited, suggesting the profound effect of membrane-bound C1q on cell proliferation. A similar effect was achieved when the cells were cultured with purified C1q or its globular heads that are known to bind to gC1qRs. When recombinant gC1qR was added to the cells, however, the effect was different. There was, instead a great increase in cell growth^16^.

Employing techniques in immunohistochemistry and immunocapture sandwich ELISA (enzyme-linked immunosorbent assay), the tissue expression of gC1qR and the blood serum of healthy controls and pancreatic cancer patients were examined. The results of the study confirmed that gC1qR is overexpressed in pancreatic adenocarcinoma when compared with healthy tissue. The serum levels of soluble gC1qR in healthy controls were noted to be 0.15 ± 0.10 ng/mL. In patients with pancreatic cancer, the levels were 0.29 ± 0.22 ng/mL and a majority of these metastatic patients displayed an increase in gC1qR serum levels as their condition worsened^92^. This indicates the significance of soluble gC1qR as a marker and possibly even an initiator of robust cellular proliferation. Another study explored the overexpression of gC1qR in MPM of 265 patients^90^. The data indicated that gC1qR overexpression was found in all types of MPM, which were proliferative compared with normal tissue, and that expression levels could be tied to the overall survival of a patient. Therefore, measuring gC1qR levels is a possible prognostic tool in the future^90^.

**Figure 4.**
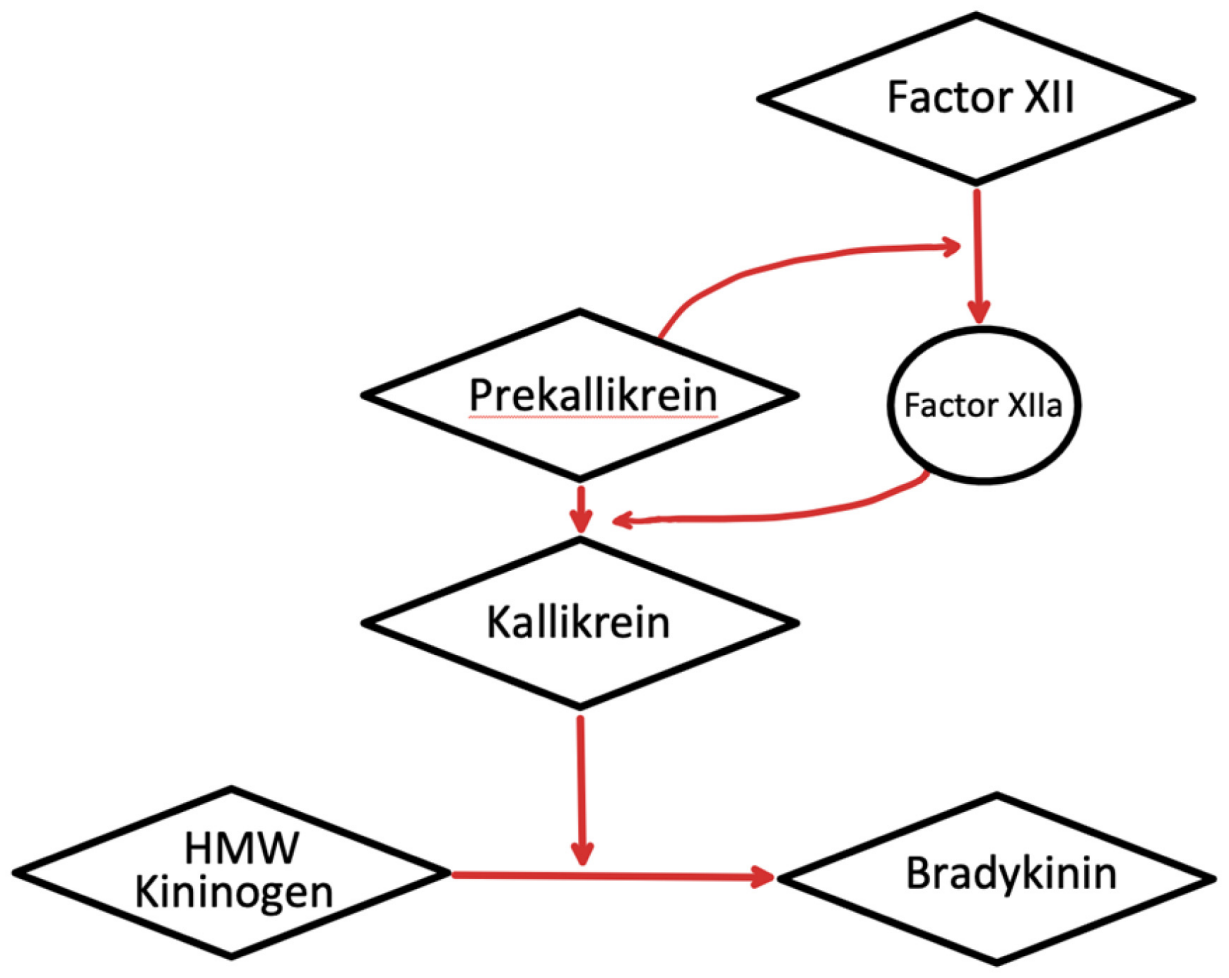
A schematic representation demonstrating the kallikrein–kinin system activation. HMW, high-molecular-weight.

It has also been observed that sites of inflammation that are characterized by the expression of gC1qR, both in the soluble form and on cell surfaces, can trigger complement activation. Since gC1qR potentially activates both the classical pathway of complement and the KKS, it can contribute to the inflammatory processes via the generation of vasoactive peptides from both the complement system and the KKS^93^. Similar to the autocatalytic activation characterized in the complement system, components of the KKS (factor XII, prekallikrein, and high-molecular-weight HK) undergo proteolytic enzyme cascade sequences which allows the generation of bradykinin^93^. Initial binding to a negatively charged cell surface causes the conversion of factor XII to XIIa, which in turn leads to the conversion of prekallikrein to kallikrein (Figure 4), and the latter is responsible for the digestion of HK to generate bradykinin^93^. Since HK binds to surface-expressed or soluble gC1qR with high affinity, the tumor cell surface as well as the TME, which is rich in gC1qR, provide a suitable milieu for the generation of bradykinin^94^. The kinin receptors such as B2K, which is constitutively expressed on a wide range of cells, and the B1K, which is inducible by IL-1β and soluble gC1qR (Figure 4), have been identified as promising targets for cancer therapy for a multitude of reasons: (a) production of kinins is a common characteristic in cancer since they are often used as autocrine factors to promote tumorigenesis, (b) kinins possess pro-angiogenic properties, (c) abnormally high concentrations of these receptors are expressed by tumor cells, and (d) components of the TME, including macrophages and DCs, are activated following activation of kinin receptors^95^.

**Figure 5.**
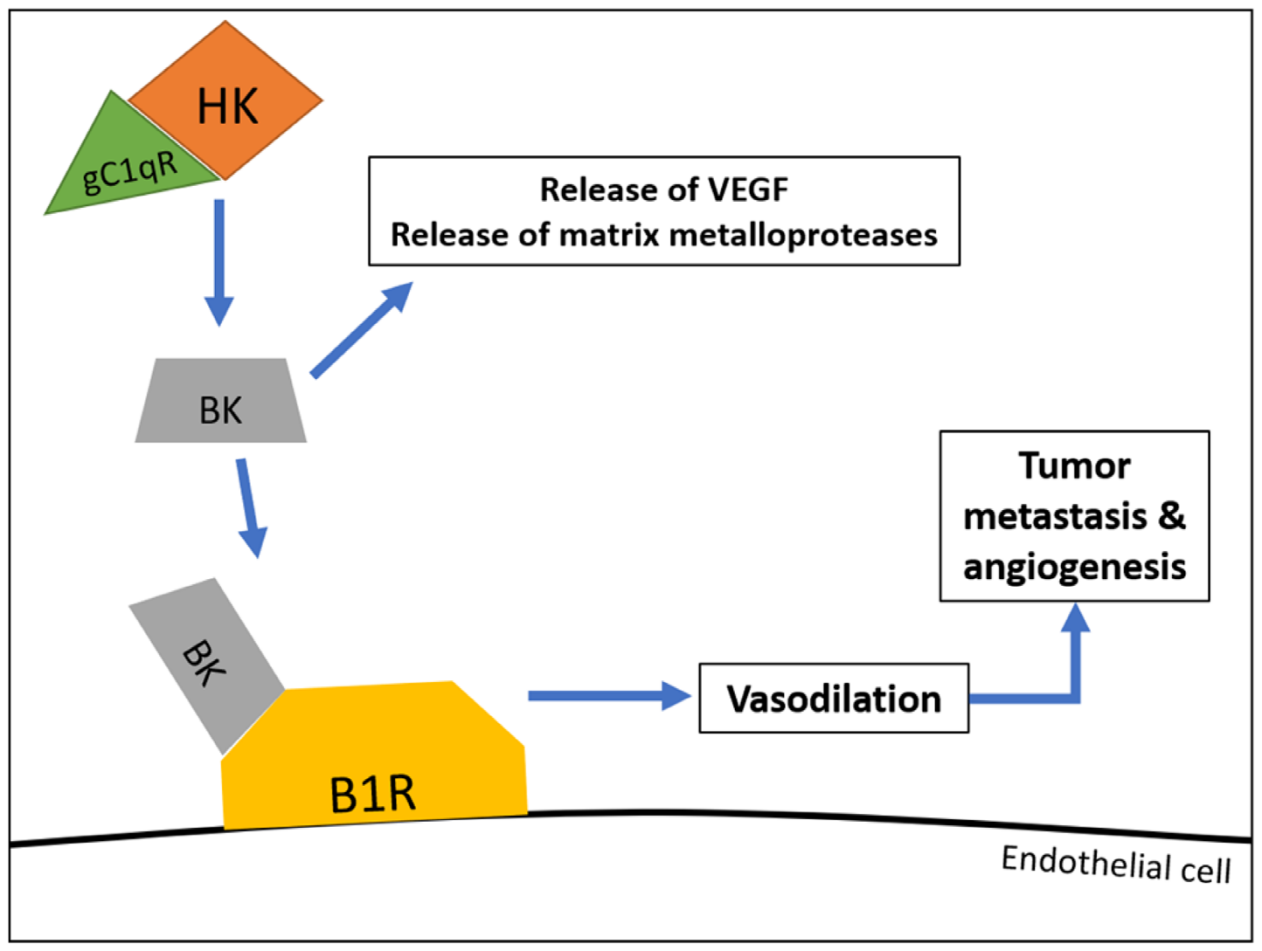
Hypothetical model demonstrating the role of gC1qR in bradykinin generation and subsequent vascular permeability. In this partial model, high-affinity binding of high-molecular-weight kininogen (HK) to gC1qR leads to bradykinin (BK) generation. Bradykinin hereafter binds to bradykinin receptor 1 (B1R) receptors found on endothelial cells, inducing vasodilation, which leads to tumor metastasis and angiogenesis. VEGF, vascular endothelial growth factor.

Although the major function of bradykinin is to induce the swelling seen in angioedema, recent studies have suggested that bradykinin is also involved in tumor metastasis and angiogenesis. Furthermore, bradykinin has been shown to directly stimulate the growth of tumors as well as induce neovascularization of tumors via release of VGEFs^96^. In addition, bradykinin has been shown to release matrix metalloproteases which facilitate tumor cell migration and invasion^96^. Therefore, if the KKS involvement is to be mitigated, then blockade of gC1qR may be a suitable option for drug design (Figure 5).

## Therapeutic potential of complement in the tumor microenvironment

The SkBr3 breast cancer studies have shown that secreted soluble gC1qR can protect the tumor cell from C1q and complement-mediated destruction. Although future investigation has to be carried out to see whether this holds true for other cancer types, it is known that many cancer cells express these proteins in the TME. Therefore, blockade of the secreted gC1qR in the TME potentially weakens tumor cell defenses and allows immune cells to dispose of the cancer cells^16^. Other complement proteins are also highly involved in the TME. Studies have found that serum levels of C3a and C5a are elevated in certain cancers and can be used as biomarkers for tumorigenic progression^97,98^. C3a and C4a can be predictive elements of a patient’s response to chemotherapy as there is a higher amount of these complement components in poor responders^99^. It has also been shown that C3aR or C5aR blockade has the ability to disrupt DC and T-cell activation and alter the effects of radiotherapy in certain carcinomas. Thus, there is growing evidence that the components of the complement system can all be good prognostic factors and even potential targets for therapy^26^.

The dual role of complement in cancer allows for potential therapeutic targets in anti-tumor studies. As discussed by Kourtzelis and Rafail^100^, a probable opportunity would be to introduce bi-specific mAbs that have the capacity to recognize two different epitopes concurrently, thereby allowing for selective neutralization of complement regulation. This would inherently permit a safe pathway for specific antibody-mediated clearance of tumor cells without causing unnecessary harm to other cells. Given that many complement proteins are essential for the proper function of the host immune response, creating therapeutic targets specifically for such components could create undesirable effects that inflict more harm through novel passages for tumor progression. However, selective neutralization of these complement components may allow for effective anti-tumor therapies, as shown by blockade of C3aR and C5aR^26^. In fact, an anti-C5aR1 antibody, IPH5401, in combination with durvalumab, is in an ongoing phase I trial for advanced solid tumors^101^. Durvalumab, a drug that blocks interaction of PD-1 and PD-L1, was included on the basis of preclinical data suggesting that the use of an anti-PD-L1 therapy with IPH5401 would reduce tumor growth, delay tumor progression, and improve overall efficacy^101^.

Indeed, the relevance of complement in carcinogenesis is becoming clear. Therefore, it will not be long before targeted therapeutic modalities are designed to block the deleterious effect of a specific complement protein.

## Conclusions

The complement system undoubtedly plays an important role in the TME. The gC1qR and C1q axis are vital elements that are potential therapeutic targets. As a modified self, a tumor cell is capable of expressing modified self-antigens that induce antibody production. These antibodies in turn can bind to the antigen target on the tumor cell, making it susceptible to recognition by C1q and destruction by complement^16^. Therefore, disarming of the gC1qR “blockade” should weaken tumor cell defenses and allow immune cells to dispose of the cancerous cells appropriately.

Various components of the complement pathway have been shown to play a role in complement activation and tumor progression. In particular, C1q has been shown in increased quantities within the TME without the subsequent presence of other vital complement proteins, indicating the relevance of C1q and its involvement within the tumor progression independent of complement activation. Several studies have suggested that in the presence of C1q, tumor progression was more rampant compared with absence of C1q, which displayed stagnant tumor growth and extended survival for cancer. Indeed, this shows that more research is needed to understand the exact role that C1q plays within the TME. In addition, it has been shown that C1q induces either pro-tumorigenic properties or anti-tumorigenic properties depending on the orientation of the C1q molecule upon binding and the specific tumor cell that it binds to. This orientation in turn depends on whether it binds to gC1qR or cC1qR on the cell surface. The multiplicity of roles that complement activation plays in the TME is increasing. It plays a role in immunosuppression through the recruitment and activation of MDSCs and is involved in neovascularization and angiogenesis, one of the notable hallmarks of cancer in which tumor cells have the ability to proliferate and metastasize through manipulative usage of nutrients and oxygen present within the environment. Complement activation in the TME also suggests that tumor-promoting inflammation is a critical component in tumor progression via the dysregulation of ILs, specific glycoproteins responsible for the recruitment of leukocytes that supervise the host immune response. Furthermore, activation of complement in the TME has been shown to prevent the activation of apoptotic components leading to inhibition of host programmed cell death and evasion of malignant cells. Therefore, the role of complement in cancer progression and survival is an area of research that has been steadily progressing albeit at a snail’s pace. Studies that ensure appropriate activation and functioning of complement proteins as well as the design of inhibitory components that restrain deleterious effects of complement are thus sorely missing.

## Abbreviations

C5aR, C5a receptor; cC1q, the collagen domain of C1q; cC1qR, receptor for cC1q; CR, calreticulin (another name for cC1qR); DC, dendritic cell; gC1q, globular head of C1q; gC1qR, receptor for gC1q; ghA, ghB, and ghC, globular heads of the A, B, and C chains of C1q; HA, hyaluronic acid; HK, kininogen; IL, interleukin; KKS, kinin–kallikrein system; mAb, monoclonal antibody; MAC, membrane attack complex; MASP, mannose-binding lectin-associated serine protease; MBL, mannose-binding lectin; mCRP, membrane-bound complement regulatory protein; MDSC, myeloid-derived suppressor cell; MPM, malignant pleural mesothelioma; PD-L, programmed death-ligand; S-face, solution face; TAM, tumor-associated macrophage; TIL, tumor-infiltrating lymphocyte; TME, tumor microenvironment; VEGF, vascular endothelial growth factor
